# Effect of self‐management intervention on prognosis of patients with chronic heart failure: A meta‐analysis

**DOI:** 10.1002/nop2.1489

**Published:** 2022-11-20

**Authors:** Chunqian Feng, Yanmei Wang, Shuang Li, Zhi Qu, Shanqing Zheng

**Affiliations:** ^1^ Institute of Chronic Disease Risks Assessment, School of Nursing and Health Henan University Kaifeng China; ^2^ School of Basic Medical Sciences Henan University Kaifeng China

**Keywords:** chronic heart failure, meta‐analysis, prognosis, self‐management

## Abstract

**Aim:**

The purpose of this study is to explore the influence of self‐management intervention on four prognostic indicators of readmission rate, mortality rate, self‐management ability and quality of life in patients with chronic heart failure.

**Design:**

A meta‐analysis.

**Methods:**

This study was selected from the related studies published from January 1999 to January 2022, and was searched by searching five databases: PubMed, Science of Website, China National Knowledge Infrastructure (CNKI), Wan Fang and Wei Pu (VIP). All standardized randomized controlled trial studies were collected, and the quality evaluation and meta‐analysis of the included literature were conducted.

**Results:**

This study included 20 randomized controlled trials involving 3459 patients with chronic heart failure. Meta‐analysis results showed that self‐management intervention could reduce the readmission rate of patients with chronic heart failure, improved self‐management ability of patients, improved quality of life, but there was no statistical significance in mortality.

## 1 BACKGROUND

Chronic congestive heart failure, referred to as chronic heart failure, is a clinical syndrome of impaired ventricular filling and ejection caused by various cardiac structural or functional diseases, and is also the terminal stage of various cardiac diseases (Welsh et al., 2018). Currently, there are 40 million patients with heart failure worldwide, including 6.2 million in the United States and over 6.5 million in China, which is expected to increase up to 45% by 2023 (Baman & Ahmad, 2020; Benjamin et al., 2019; Hu et al., 2019). According to statistics, the readmission rate for heart failure patients in the United States in 2013 was 22.9%, and in China, research also showed readmission in patients with heart failure rate as high as 58.4% (Huang et al., 2020; Suter et al., 2014; Wang et al., 2018). Despite the continuous improvement of drug therapy and non‐drug therapy, the overall control situation remains unsatisfactory, and the mortality rate of heart failure is still at high level. An epidemiological survey found that heart failure accounted for one in 8.6 death certificates in the United States. Worldwide, 17%–45% of patients with chronic heart failure die in the first year after admission, with most dying in 5 years (De Sutter et al., 2015; Ponikowski et al., 2014). Chronic heart failure has become a serious disease affecting public health and quality of life due to its high prevalence, readmission and mortality rates (Snipelisky et al., 2019). How to actively treat chronic heart failure, reduce the rate of readmission and mortality of patients, and improve the quality of life of patients has become an important task in clinical work. Studies found that a lack of self‐management ability is not only the key factors affecting the prognosis of patients with chronic heart failure, and is also the main reason for the patients with chronic heart failure readmission (Hägglund et al., 2015; Mariko et al., 2019; Wu et al., 2012. Therefore, we hypothesized that self‐management interventions with patients can reduce readmission rate and mortality, improve self‐management ability, and thus improve their quality of life.

Self‐management is the core concept of chronic heart failure management (Wagner et al., 2001). Creer (2000) showed that self‐management of chronic heart failure mainly includes six aspects: target selection, information collection, information judgement, decision‐making, action taking and self‐feedback. Subsequently, Clark et al. (2014) conducted an in‐depth study on self‐management of chronic heart failure, believing that self‐management of chronic heart failure refers to the observation, judgement and response of internal factors such as knowledge, attitude, feeling and belief, and external factors such as role expectation and social support, so as to adopt disease management strategies to achieve the desired goals. In 2002, Barlow et al. (2002) defined self‐management as the ability of patients to manage symptoms, treatment, physiological and psychosocial changes, and make lifestyle changes developed in the process of coping with chronic diseases. Kang et al. (2012) defined self‐management of patients with chronic heart failure by Rodgers evolutionary concept analysis as a series of self‐regulation behaviours effectively adopted by patients in the process of coping with heart failure, such as through the knowledge of disease and improve the self‐efficacy, seek support and specific behaviour (lifestyle adjustment and symptom management behaviour) and self‐perception (internal and external factors and self‐adjusting), to control the disease influence ability, to achieve a satisfactory quality of life. The World Health Organization (WHO) defined quality of life as the experience of individuals in different cultures and value systems on their living conditions related to their goals, expectations, standards and concerns (Orley & Kuyken, 1994). Studies have found that there is a correlation between quality of life and self‐management, and the two are mutually causal. Quality of life is one of the influencing factors of self‐management, and quality of life can be regarded as an outcome of self‐management (Jaarsma et al., 2021; Zhang & Li, 2009).

At present, the self‐management level of chronic heart failure patients is mostly below the medium level. Riegel, Driscoll, et al. (2009) and Riegel, Moser, et al. (2009) investigated the self‐management behaviour of 2082 patients with chronic heart failure in two developed countries, the United States and Australia, and two developing countries, Mexico and Thailand, results showed that about 70% of patients with chronic heart failure had low self‐management level. In the United States, 52% of patients achieved the self‐management ability score, but in Thailand was just 5%. Li et al. (2020) investigated 6,124 patients with chronic heart failure from 102 hospitals in 16 provinces, municipalities and autonomous regions in China, and found that the total score of self‐management of patients with chronic heart failure was 49.00 (43.00, 54.00). According to the above studies, improving the self‐management ability of patients with chronic heart failure has become one of the clinical nursing problems that need to be solved.

In 2009, Riegel, Driscoll, et al. (2009) Riegel, Moser, et al. (2009) summarized the measures to improve the self‐management behaviour of patients with chronic heart failure mainly as the self‐monitoring of symptoms change, weight management, diet management, restriction of sodium and water intake, rational drug treatment, regular exercise and lifestyle adjustment. There are various ways to implement self‐management intervention. Self‐management intervention is mainly implemented through telephone, short message, network software, etc. In China, health education, follow‐up, telephone, we‐chat and other methods are used to guide patients with chronic heart failure to adjust their diet structure and lifestyle, teach patients to monitor their condition and improve their self‐management ability and quality of life (Huo et al., 2016; Jonkman et al., 2016; Marikoet al., 2019; Scott et al., 2004; Tung et al., 2013; Wang et al., 2017). Otsu and Moriyama (2011) found that self‐management intervention achieved good effect on improving the health status of patients with chronic heart failure. Wu (2012) also found that, compared with the control group, self‐management intervention could reduce the readmission rate and mortality rate of patients with chronic heart failure, and improved their self‐management ability and quality of life. However, Darren et al. (2006) showed that despite self‐management education for patients with chronic heart failure, there was no significant difference in patient readmission rates and mortality compared with conventional care. Therefore, we hope to clarify the influence of self‐management intervention on the four prognostic indicators of readmission rate, mortality rate, self‐management ability and quality of life of patients with chronic heart failure by searching literature, so as to arouse the attention and consideration of scholars. It provides a more scientific basis for improving patients' self‐management ability and quality of life and reducing patients' readmission rate and mortality rate.

## 2 RESEARCH QUESTIONS

We aimed to investigate the effects of self‐management interventions on four prognostic indicators of readmission rate, mortality, self‐management ability and quality of life in patients with chronic heart failure.

## 3 METHODS

### 3.1 Eligibility criteria and outcome variables

This study was carried out according to the inspection system reporting guidelines proposed in the statement of Preferred Reporting Items for Systematic Reviews and Meta‐Analyses (PRISMA; Vrabel, 2009). In order to select relevant research, a systematic literature search was conducted based on PICO‐SD (population, intervention, comparison, results, research design). The population of this study (P) is chronic heart failure patients over 18 years old, and the clinical diagnosis was consistent with the diagnostic criteria of chronic heart failure in the European society of cardiology guidelines for the diagnosis and treatment of acute and chronic heart failure in 2016 and the Chinese guidelines for the diagnosis and treatment of heart failure in 2018. Guidelines included a history of coronary heart disease, hypertensive heart disease, dilated cardiomyopathy and other basic cardiovascular diseases. Symptoms such as dyspnoea, fatigue and lower limb oedema during rest or exercise; cardiac colour ultrasound suggests cardiac enlargement, etc. New York Heart Association (NYHA) diagnostic cardiac function grades ii to IV; Left ventricular ejection fraction (LVEF) > 30%, and serological detection of NT‐PRO BNP <5,000 pg/ml (Ponikowski et al., 2016; Wang & Liang, 2018; Yancy et al., 2013); Interventions (I) are self‐management interventions, which mainly covers self‐management education, follow‐up, use of telephone, SMS or Internet software, etc.; Control group (C) included patients receiving routine care, including diet, disease knowledge, general care such as exercise, discharge guidance and routine follow‐up, etc.; Outcomes (O) readmission rates, mortality rates, quality of life and self‐management; Study design (SD) was a randomized controlled trial.

### 3.2 Literature retrieval strategy

In this study, PubMed, Science of Website, China National Knowledge Infrastructure (CNKI), Wan Fang and Wei Pu (VIP) were searched, and the relevant studies published from January 1999–January 2022 were selected. We used the following keywords to search for literature: (“self‐management” or “self‐management program” or “self‐management intervention”), (“chronic heart failure” or “heart failure” or “congestive heart failure”). The study has no language restrictions.

### 3.3 Inclusion and exclusion criteria of literature

The criteria of literature inclusion should be (1) randomized controlled trial (RCT); (2) the subjects were patients aged 18 or above who met the diagnostic criteria of chronic heart failure; (3) the experimental group received self‐management intervention on the basis of routine nursing, such as family interview, telephone supervision, health education and family remote interventions. The control group only received routine nursing care. (4) the indexes included in the literature include, but are not limited to, any one or more of the four indexes: readmission rate, mortality rate, quality of life and self‐management ability.

The exclusion criteria are: (1) review or meta‐analysis; (2) unable to obtain full‐text literature; (3) documents that cannot obtain valid data; (4) literature with inconsistent outcome indicators.

### 3.4 Literature screening and data extraction

In this study, two researchers independently read the titles and abstracts of literature to determine whether they meet the inclusion criteria. During the process of literature screening, the collected literatures were downloaded and retrieved through Endnote, and duplicate literatures were deleted. By reading the topics and abstract, eliminated the papers that are obviously inconsistent with the research content, object, method and achievement index of these papers, downloaded the papers that meet the requirements for reading, and checked whether they meet the inclusion criteria. Disputes can be resolved through consultation or a third party. We extracted and sorted out the following data from each selected research: first author, year, sample size, intervention measures, intervention time and result indicators.

### 3.5 Literature quality evaluation

This study referred to the deviation risk assessment tool of RCT in The Cochrane Manual of Systematic Evaluation 5.1.0. The quality of the included literature was evaluated mainly based on seven aspects: generation of random sequence, hidden distribution, whether to apply blinds to implementers and participants, blinds in outcome evaluation, integrity of outcome data, selective reporting, and whether there are other biases (Wang, 2019). The evaluation level of each content is divided into high risk, low risk and ambiguity. According to the possibility of result deviation, the literature quality grade in this study was divided into A, B and C, where A is high‐quality literature, B is medium‐quality literature and C is low‐quality literature.

### 3.6 Statistical methods

This study evaluated the effects of self‐management interventions on four outcome indicators of readmission, mortality, self‐care ability and quality of life in patients with chronic heart failure. We analysed self‐management ability and quality of life as continuous variables and hospital readmission and mortality rates as dichotomies. Standardized mean difference (SMD) was used for continuous data, and relative risk (OR) was used for dichotomous data. Each effect size was expressed as a 95% confidence interval (CI), and *p* < .05 was considered statistically significant. We used the *I* ^2^ test to quantify the heterogeneity of the study. If *p* < .05 and or *I* ^2^ > 50%, random effects model will be used, otherwise fixed effects model will be used. In the meta‐analysis of each outcome, sensitivity analysis was performed. We evaluated the symmetry of funnel plots using the Egger test and defined significant publication bias as a *p* value < .1. Stata software (version 16.0) was used for all statistical analysis (Stata Company).

## 4 RESULTS

### 4.1 Search results

**FIGURE 1:**
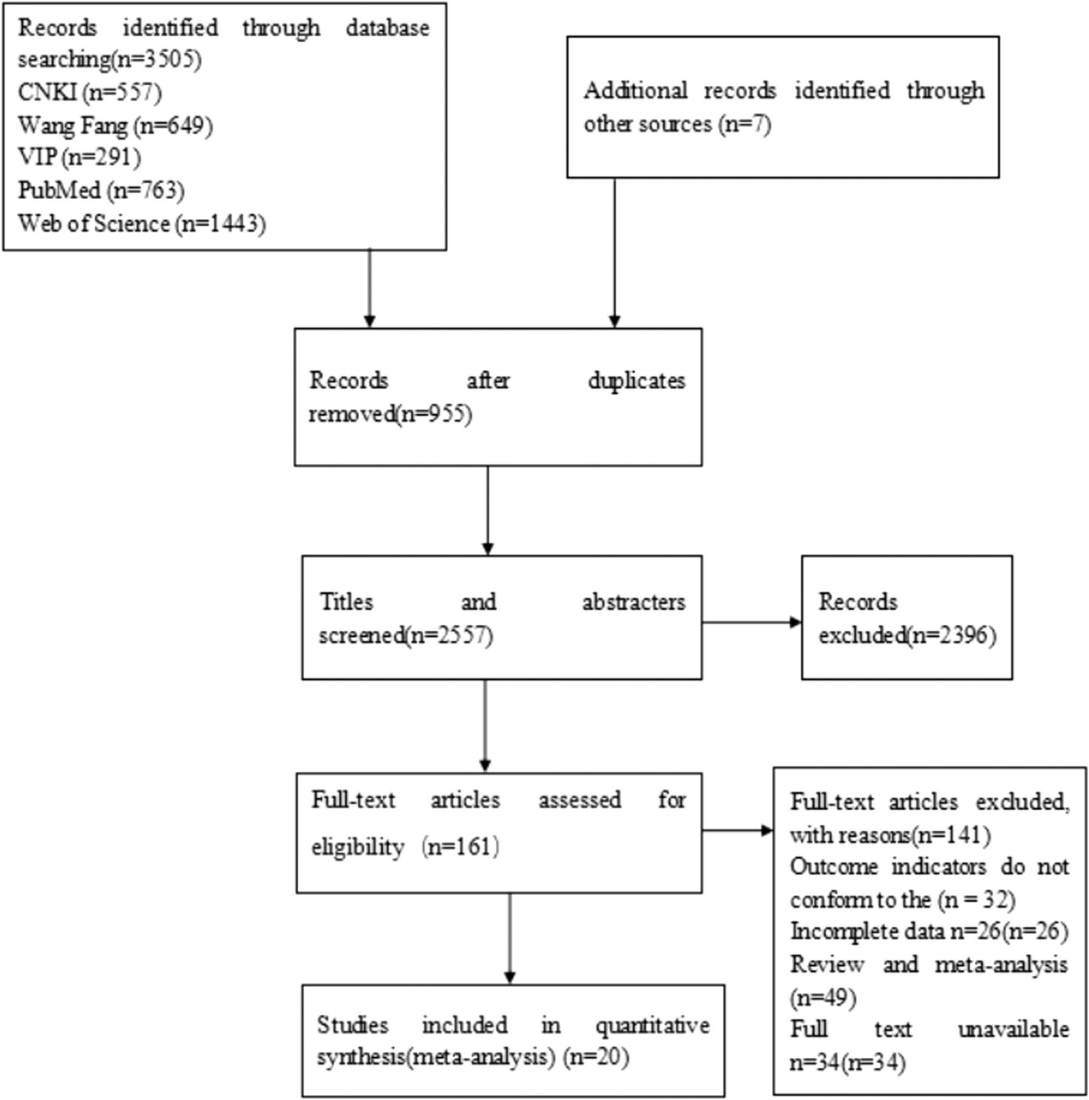
Flow chart of literature search and study selection

In this study, 3,512 related literatures were searched preliminarily, and 955 duplicated literatures were deleted. After reading the titles and abstractions, 2,396 literatures which were obviously inconsistent with the research content, research object, research method and achievement index of this paper were deleted, and then 161 literatures were entered into the next screening. After reading the full text, 20 articles were included after 141 articles with incomplete data, inconsistent outcome indicators, meta‐analyses and reviews, and no full text was available (Cockayne et al., 2014; Darren et al., 2006; Dorsch et al., 2021; Du, 2018; Dunagan et al., 2005; Fu & Gu, 2016; Jaarsma et al., 1999; Jiang et al., 2021; Li et al., 2018; Powell et al., 2010; Mariko et al., 2019; Nucifora et al., 2006; Smeulders et al., 2010; Sun et al., 2019; Wang et al., 2017; Wu, 2012; Xu et al., 2016; Yang, 2018; Yang et al., 2019, 2021). See Figure 1 for the literature screening process, and see Table 1 for the basic characteristics of the included literature.

**TABLE 1:** Basic features of the included literature

Authors	Year	Sample		Intervention measures		Intervention duration (months)	Outcome indicators
		Experimental group	Control group	Experimental group	Control group		
Wang	2017	30	30	Self‐management education and support based on mobile health mainly relies on the platform to release health information, monitor and feedback the condition, provide peer support and other information	Usual care	6	SCHFI, MLWHF, Readmission rate
Xu	2016	50	48	Hospital on the day of one‐on‐one communication with patients, health education plan, implementation of self‐management support strategy (symptoms psychological support, management support, management support and drug management), regular follow‐up	Usual care	6	Readmission rate, mortality rate
Du	2018	38	32	Standardization of self‐management measures, including the reasonable medication guide, sports rehabilitation guidance, train the ability of self‐management, health education, nutritional guidance, psychological counselling, and regular follow‐up	Usual care	12	Self‐management level scale
Yang	2019	50	50	Self‐management education, the establishment of health records, explaining the knowledge of the disease, emphasizing the importance of self‐management, the development of self‐management programs, including symptoms, behaviour, mood and knowledge of self‐management and follow‐up	Usual care	12	MLWHF
Li	2018	100	100	Community outpatient follow‐up intervention, self‐management intervention (symptom management, medication, diet limit water, active rest, stress management and support for education module), and self‐management training for the patient	Usual care	12	Readmission rate, mortality rate
Yang	2021	102	102	Self‐management education mainly includes face‐to‐face education and guidance, covering psychological guidance, life management, disease and medical management, daily activities and self‐management ability, etc. Regular forums are held for communication, self‐management education plans are formulated, knowledge is regularly pushed through We‐chat platform, and patients' knowledge is timely fed back	Usual care	6	MLWHF, Self‐management level scale
Wu	2012	30	32	Self‐management model theory as the instruction, make self‐management measures, including strengthening education, train the ability of patient self‐management, exercise guidance, build system of follow‐up and psychological counselling	Usual care	10	Self‐management level scale, readmission rate, mortality rate
Yang	2018	40	40	Self‐management mode, including hospital management, daily management guidance, to set up the patients with heart failure patients BBS and multimode continuance management, follow‐up	Usual care	6	SCHF, mortality rate, readmission rate
Fu	2016	63	63	Self‐management program mainly includes the patients self‐identify key symptoms, patients well management and daily self‐discharge follow‐up and family visit	Usual care	6	SCHFI, readmission rate
Cockayne	2014	95	165	Self‐management plan contains six structured one‐on‐one education courses, and regular monitoring of signs and symptoms, clinical assessment and referral	Usual care	12	Readmission rate
Darren	2006	59	64	Self‐management education, which emphasizes daily weight measurement, self‐adjustment of diuretic dose, and symptom recognition and response, provides patients with image‐based educational materials, digital scales, and regular telephone follow‐up visits	Usual care	12	Readmission rate, mortality rate
Dorsch	2006	42	41	Daily self‐monitoring using mobile apps, providing health indicators and education to promote self‐management, active self‐monitoring prompts through mobile app push notifications, and completing an 8‐question survey in the app	Usual care	3	MLWHF, SCHFI, Readmission rate
Dunagan	2005	76	75	Regular telephone monitoring to improve patients' self‐management skills, appropriate dietary guidance and adherence to guidelight‐based treatment, use of standardized screening tools to monitor changes in disease, and increase and adjust the frequency of phone calls after discharge	Usual care	12	Readmission rate, mortality rate
Jiang	2021	49	56	Self‐management intervention plan mainly includes the patient education, self‐management package, and regular home visits	Usual care	6	MLWHF
Powell	2010	451	451	Email tip hf single heart failure management of the basic elements, namely the main content including drugs, weight management, salt restriction, moderate physical activity and stress management, personal management skills	Usual care	12	Readmission rate
Nucifora	2006	99	101	Structured patient education face to face, the main content for the cause of heart failure, and identification of worsening heart failure symptoms, sodium restrictions and the effect of treatment with drugs, body fluids, and the importance of weight control, physical activity and completely give up drinking and smoking, increase call after discharge frequency, encourage patients in disease progression or when in doubt call and regular follow‐up	Usual care	6	MLWHF
Smeulders	2010	186	131	Structured self‐management program mainly includes modelling and social skills, to explain symptoms, persuade four strategies to improve self‐efficacy expectations	Usual care	12	EHFScBS, KCCQ
Sun	2019	50	50	Self‐management plan mainly covers the primary disease‐related knowledge, disease pathogenesis, and treatment objectives, guidelines, exercise, nutritional guidance, prevention of acute attack, nursing after discharge based on Internet platform monitoring patients and to answer questions, send a message to the patient through the APP or WeChat and regular follow‐up	Usual care	6	SCHFI
Jaarsma	1999	84	95	Structured education programs cover identifying warning symptoms of worsening heart failure, sodium limitation, fluid balance and compliance, social interaction, sexual function, and follow‐up or home visits after discharge	Usual care	9	Readmission rate
Mariko	2019	20	19	Face‐to‐face interviews mainly cover the aetiology and self‐management knowledge of the disease, the management of symptoms and signs, diet, especially the restriction of intake of water and sodium, drug management, activity guidance, stress guidance, etc., the establishment of short‐term goals and long‐term goals, monthly assessment and revision of action plans	Usual care	12	Readmission rate, mortality rate

Abbreviations: EHFScBS, European Heart Failure Self‐Care Behaviour Scale; KCCQ, Kansas City Cardiomyopathy Questionnaire; MLWHF, Minnesota living with heart failure questionnaire; SCHFI, Self‐Care of heart failure index.

### 4.2 Quality evaluation of literature

According to Cochrane bias risk assessment, 20 RCTs were included in this study and relevant risk biases included in the quality assessment literature were classified (Wang, 2019). Among them, seven papers were rated as Grade A, and 13 papers were rated as Grade B, and the results are shown in Table 2.

**TABLE 2:** Quality assessment of the included literature

Authors	Random sequence generation	Allocation concealment	Blinding of participants and personnel	Blinding of outcome assessment	Incomplete outcome data	Selective reporting	Other bias	Quality grade
Wang	√	√	?	√	√	√	√	A
Xu	?	?	?	?	√	?	?	B
Du	×	×	?	?	√	√	√	B
Yang	?	?	?	?	√	?	?	B
Li	√	√	?	√	√	√	√	A
Yang	×	×	?	?	√	?	?	B
Wu	√	√	?	?	√	√	√	B
Yang	√	√	?	?	√	√	?	B
Fu	√	√	?	?	√	?	?	B
Cockayne	√	√	?	?	√	√	√	A
Darren	√	√	?	√	√	√	√	A
Dorsch	√	√	?	?	√	?	?	B
Dunagan	?	?	?	?	×	?	×	B
Jiang	√	√	?	√	√	√	√	A
Powell	√	√	√	√	√	√	√	A
Nucifora	×	×	?	?	√	?	?	B
Smeulders	√	√	√	?	√	√	?	B
Sun	√	√	?	√	√	?	?	B
Jaarsma	√	√	?	?	√	√	√	A
Mariko	√	√	?	?	√	?	?	B

*Note*: √ = Low risk; × = high risk; ? = unclear.

### 4.3 Meta‐analysis results

#### 4.3.1 Impact on readmission rate

**FIGURE 2:**
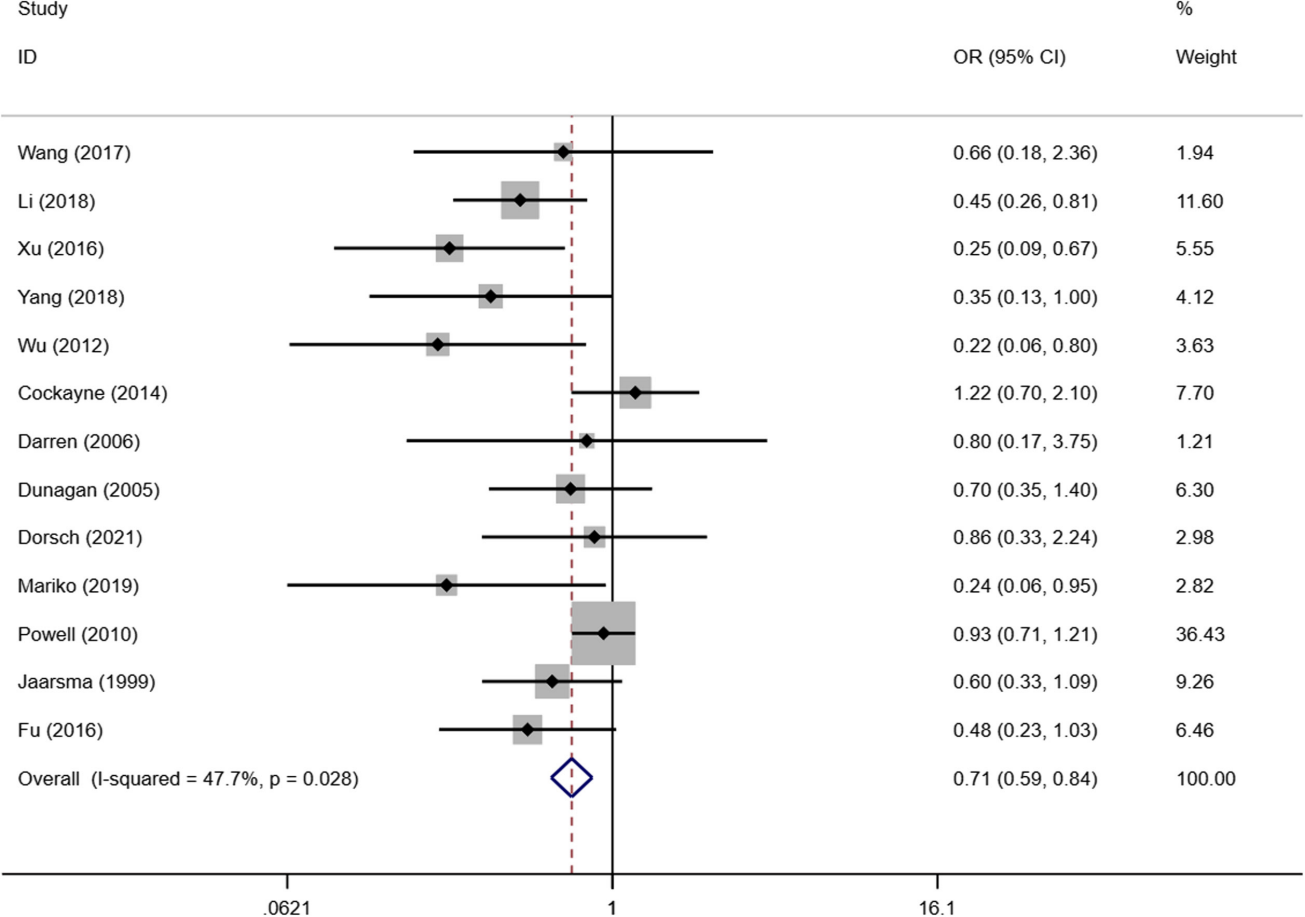
Forest plot of the effect of self‐management intervention on the readmission rate of patients with chronic heart failure

In this study, we included 13 studies evaluating the impact of self‐management interventions on readmission rates in2363 patients with chronic heart failure (Cockayne et al., 2014; Dorsch et al., 2021; Dunagan et al., 2005; Darren et al., 2006; Fu & Gu, 2016; Jaarsma et al., 1999; Li et al., 2018; Powell et al., 2010; Mariko et al., 2019; Wu, 2012; Xu et al., 2016; Wang et al., 2017; Yang, 2018). The inter‐study heterogeneity test results were *I* ^2^ = 47.7% *p* = .028, indicating the heterogeneity of this study. Therefore, we used a fixed‐effects model for meta‐analysis, which showed that self‐management interventions reduced readmission rates of patients with statistically significant differences [OR = 0.706, 95% CI (0.593, 0.841), *p* < .001] (Figure 2). Through sensitivity analysis, we found that excluding Lynda study, deletion of other studies had little effect on heterogeneity results. However, after excluding Powell study, we found that heterogeneity decreased from 47.7%–33.5%, which may be due to the large sample size of this study, so the weight is large, which significantly affects the effect of merger.

#### 4.3.2 Impact on mortality

**FIGURE 3:**
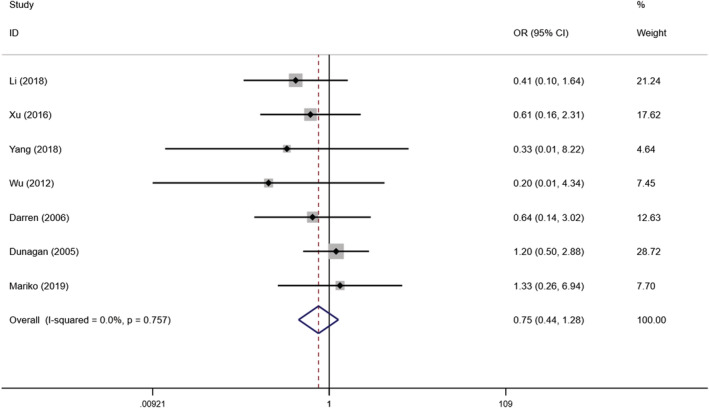
Forest plot of the influence of self‐management intervention on mortality of patients with chronic heart failure

In this study, we identified seven papers on the effect of self‐management interventions on death in 741 patients with chronic heart failure (Dunagan et al., 2005; Fu & Gu, 2016; Li et al., 2018; Mariko et al., 2019; Wu, 2012; Xu et al., 2016; Yang, 2018). The heterogeneity test result between studies was *I* ^2^ = 0% *p* = .757, indicating that there was no heterogeneity between studies. We performed a meta‐analysis using a fixed‐effects model that showed no effect on patient mortality from self‐managed interventions and there was no significant difference in mortality between the trial and control groups. In other words, self‐management interventions did not reduce mortality [OR =0.753, 95% CI (0.444, 1.277), *p* = .293] (Figure 3). By sensitivity analysis, we found that deleting any study had little effect on the results.

#### 4.3.3 Impact on self‐management ability

**FIGURE 4:**
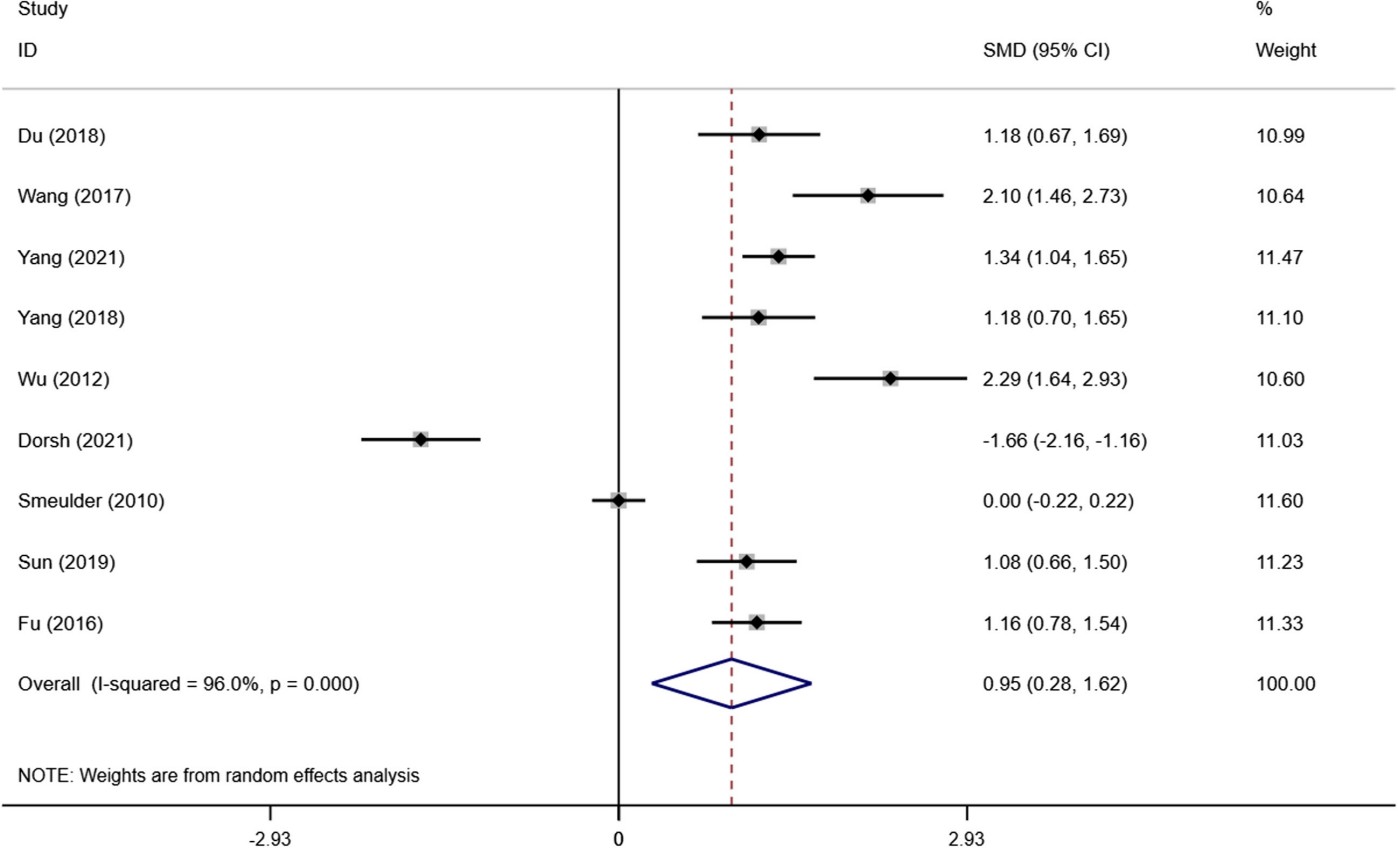
Forest plot of the influence of self‐management intervention on the self‐management ability of patients with chronic heart failure

In this study, we identified nine articles on the effect of self‐management interventions on self‐management in 1101 patients with chronic heart failure (Dorsch et al., 2021; Du, 2018; Fu & Gu, 2016; Smeulders et al., 2010; Sun et al., 2019; Wang et al., 2017; Wu, 2012; Yang, 2018; Yang et al., 2021). The heterogeneity test result between studies was *I* ^2^ = 96% *p* < .0001, indicating that there was great heterogeneity among studies. We used a random‐effects model for a meta‐analysis. The results showed that self‐management intervention could improve patients' self‐management ability, and the difference was statistically significant [SMD = 0.949, 95% CI (0.278, 1.620), *p* = .006] (Figure 4). By sensitivity analysis, we found that deleting any study had little effect on the results.

#### 4.3.4 Impact on quality of life

**FIGURE 5:**
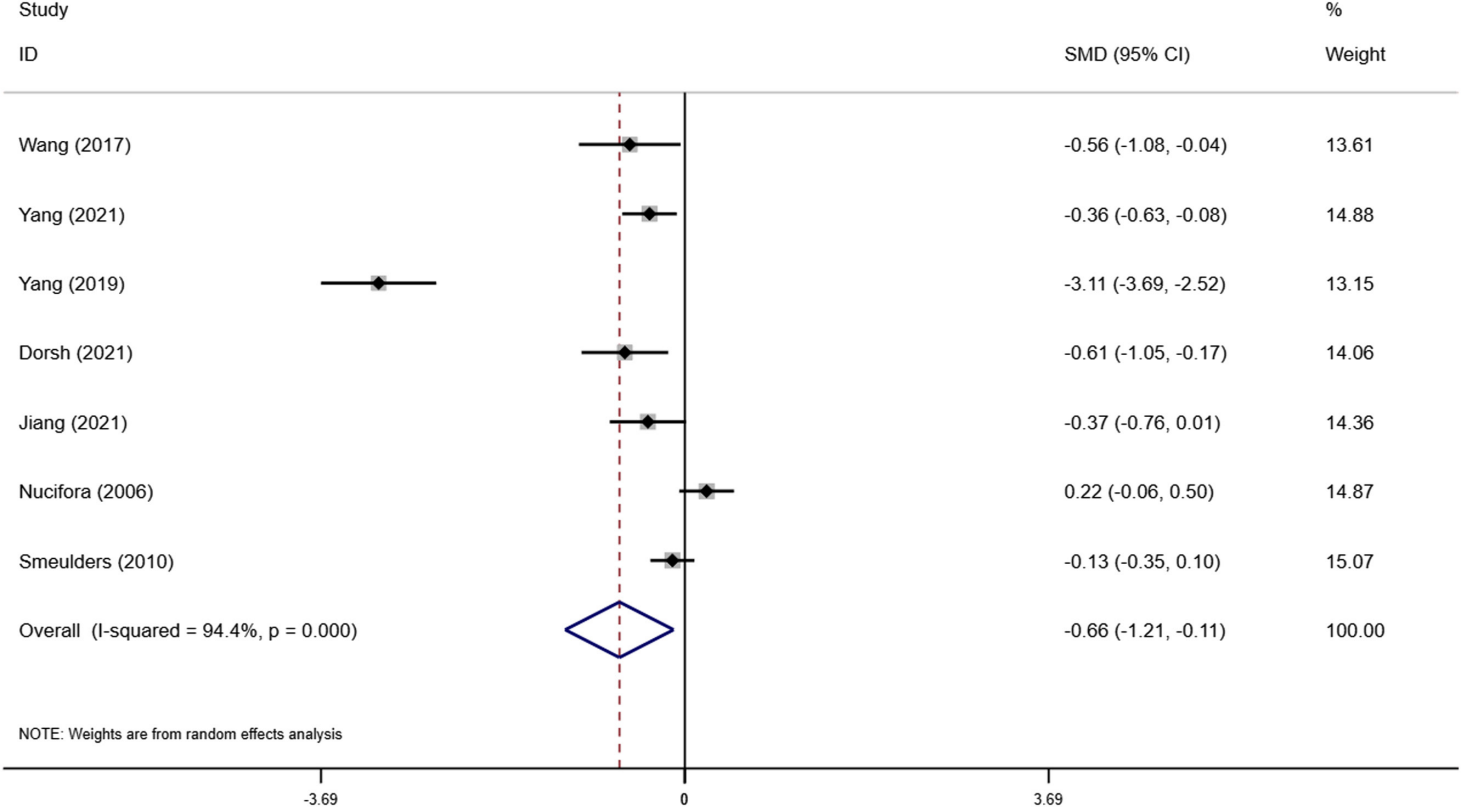
Forest plot of the influence of self‐management intervention on the quality of life of patients with chronic heart failure

In this study, we identified seven articles examining the effects of self‐management interventions on quality of life in 1069 patients with chronic heart failure (Dorsch et al., 2021; Jiang et al., 2021; Nucifora et al., 2006; Smeulders et al., 2010; Wang et al., 2017; Yang et al., 2019, 2021). The heterogeneity test results between studies were *I* ^2^ = 94.4%, *p* < .0001, indicating high heterogeneity among studies. Therefore, we performed a meta‐analysis using random‐effect wooden boxes, and the results showed that self‐management interventions significantly improved patients' quality of life, with statistically significant differences [SMD = −0.664, 95% CI (−1.214, −0.113), *p* = .018] (Figure 5). Through sensitivity analysis, we excluded Yang and Nucifora's two studies and found that heterogeneity decreased from 94.4%–27%, which may be related to the age, education level or compliance of the subjects.

#### 4.3.5 Publish the assessment results of bias risk

**FIGURE 6:**
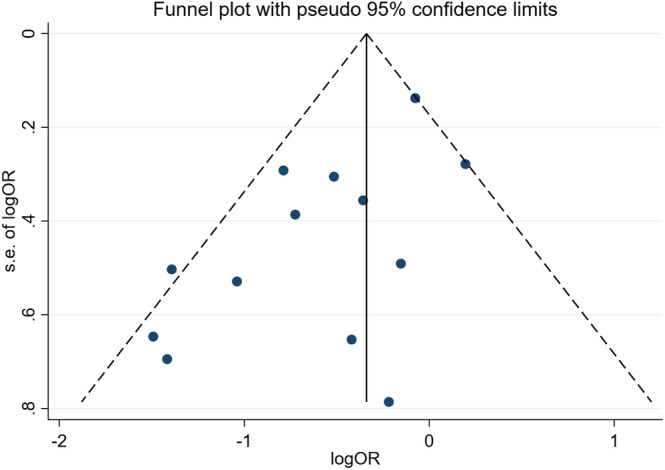
Publish bias graphs

Funnel plots were drawn for readmission rate outcome indicators of the 13 included literature (Cockayne et al., 2014; Darren et al., 2006; Dorsch et al., 2021; Dunagan et al., 2005; Fu & Gu, 2016; Jaarsma et al., 1999; Li et al., 2018;Powell et al., 2010; Mariko et al., 2019; Wu, 2012; Xu et al., 2016; Wang et al., 2017; Yang, 2018), and publication bias risk was calculated by Egger, with logor values of the included study indicators as abscissa and SE (Logor) values as ordinate. The results showed that the two sides of funnel plot were asymmetric, *t* = −2.92, *p* = .014,95% CI [−3.10, −0.44], suggesting publication bias. (Figure 6).

## 5 DISCUSSION

The results of this study suggested that self‐management interventions can reduce patient readmission rates compared to conventional care and this is consistent with the results of Gandhi et al. (2017). Studies have found that patients with chronic heart failure have poor self‐management behaviours in drug therapy, weight monitoring, diet control, exercise therapy and emotion regulation, which leads to high readmission rates and mortality rates in patients with chronic heart failure (Fu et al., 2015; Lorig & Holman, 2003). Therefore, we believed that it is necessary to help patients establish and maintain healthy self‐management behaviours in order to improve patient outcomes. In 2001, the American College of Cardiology (AHA) recommends developing a self‐management program for chronic heart failure, which aims to provide patients with relative educational materials and guidance, to help them improve their disease knowledge and symptom management skills (Riegel, Driscoll, et al., 2009; Riegel, Moser et al., 2009). At present, the methods of self‐management intervention include health education, telephone, email, monitoring and management of Internet application software and family follow‐up. The implementation of health education can be divided into two stages: in‐hospital and out‐hospital. During the patient's hospitalization, medical staff communicated with the patient face to face, evaluated the main existing problems, and formulated the corresponding self‐management education plan according to the actual situation of the patient, this plan covers the content is widespread, mainly summarized as symptom management, behaviour management, drug management and psychological support four aspects (Dorsch et al., 2021; Fu & Gu, 2016; Smeulders et al., 2010; Wang et al., 2017; Yang et al., 2021). Medical staff by using self‐management brochures, heart failure symptoms suggest education material such as single and self‐management package, and regularly carry out symposium, discussion seminars, etc., to explain the main cause of disease in patients with symptoms and acute prevention knowledge, told to take right way of life behaviour (quit alcohol, maintaining sleep), limit sodium intake, regular monitoring of water weight and the importance of appropriate physical exercise for prognosis of disease, emphasize comply with medication is the key to the treatment, do not stop taking medicine, adjust the dose of medicine, let patients know about the effect of drugs, dosage, time and adverse reactions. At the same time, pay attention to the psychological changes of patients, inform patients of the adverse reaction of negative emotions to the prognosis of the disease, encourage patients to speak out their main views on the disease and psychological pressure, and provide relaxation skills, diversion and social support measures to adjust psychological pressure. After discharge from hospital, medical personnel can use telephone contact, WeChat, E‐mail or mobile application software to realize the monitoring and management of patients, determine the frequency of using the equipment and continue to provide guidance and counselling for patients, encourage patients to provide feedback on the effect of medical staff, and provide solutions and methods for their existing problems or doubts; In addition, regular follow‐up for patients, to evaluate the current status of self‐management behaviour of the patients with self‐management behaviour and encourage have let it continue, helping patients to find the reasons for failing to realize self‐management behaviour, help patients to find relevant solutions, fully improve the patients’ self‐management ability, help them to improve the quality of life. At the same time, teaching patients to recognize the precursors of disease change or deterioration can enable patients to seek treatment as early as possible and avoid readmission, thus reducing the readmission rate of patients (Chen et al., 2015; Cockayne et al., 2014; Fu & Gu, 2016; Wang et al., 2017; Xu et al., 2016).

Data showed that the 5‐year mortality rate of patients with chronic heart failure is 52%, and the survival rate is even lower than that of malignant tumours (Go et al., 2013; Stewart et al., 2001). In the latest study, Wang et al. (2021) found that the mortality rate of patients with chronic heart failure reached 46.1% during the 5‐year follow‐up, which was still high although it was down from the previous period. The results of this study indicate that self‐management interventions did not reduce patient mortality compared with conventional care. However, this result is contrary to the conclusion of Ditewig et al., 2010. We considered that the reasons for the absence of positive results might be related to the patient's own physical condition and the compliance of disease treatment. Some patients may be complicated with multiple comorbidities such as hypertension and diabetes, while the management of self‐management intervention diseases may mostly focus on heart failure, and the management of other diseases is less involved. At the same time, it is also related to the treatment compliance of patients, which may be affected by the degree of education, understanding of the disease or familiarity with smart devices, so that the implementation effect of self‐management intervention measures may be different. Therefore, it is suggested to carry out targeted personalized and specific self‐management interventions for different population characteristics in the future, so as to observe the impact on mortality.

The results of this study indicated that self‐management intervention can improve the patient's self‐management level, and improve their quality of life. Relevant studies have found that the self‐management level of most patients with chronic heart failure is below the middle level, this may be related to the patient's age, education level, cognition of the disease and lifestyle (such as lack of recognition and prevention of the symptoms and signs of the disease, lack of knowledge of sodium intake, etc.), treatment compliance, and the influence of anxiety, depression and adverse emotions (He, 2013). Poor self‐management may increase the number of acute exacerbations, readmission frequency, economic stress and psychological stress in patients with chronic heart failure, thus affecting the quality of life. As a new strategy of tertiary prevention and health education of chronic diseases, self‐management plays an important role in improving patients’ self‐management ability and quality of life. Improving patients’ self‐management ability is also an important measure to reduce readmission rate and improve quality of life (Wu et al., 2012). We found that the self‐management intervention on patients with health education and monitoring management, let the victim to recognize their own disease harm, characteristics, causes, treatment and curative effect, learn self‐management on the importance of the development and prognosis of disease, make better cooperate with treatment, help them establish the confidence of conquer disease, Patients can be more proactive in improving their self‐management, thus improving their quality of life (Dorsch et al., 2021; Jiang et al., 2021; Wang et al., 2017; Yang et al., 2019, 2021). Although our results showed that a self‐management intervention can improve the patient's quality of life, but how to improve and maintain the quality of life in clinic is still the problem of modern nursing, as a result, the researchers in the self‐management intervention, need to pay attention to in the process of implementation of deficiencies and difficulties, find a new target to improve and maintain quality of life, to develop more beneficial and targeted interventions.

## 6 LIMITATIONS

Our current research has several advantages. Firstly, compared with the original research, our sample size is larger and our statistical ability is stronger, so our results are more reliable. Secondly, we evaluate multiple outcome indicators of the prognosis of patients with chronic heart failure. At the same time, we should also admit some limitations of this research. Firstly, the literature included in this study were collected for secondary analysis, which would produce artificial bias when conducting methodological evaluation. Secondly, none of the included studies was blind, so there was selective bias to some extent. The included population was a mixed Chinese and Western population, with regional cultural differences and clinical heterogeneity to a certain extent. Finally, patients' age, educational level, compliance, intervention methods, duration and other factors will have an impact on the intervention effect. Further large‐sample randomized controlled trials are needed to explore the impact of these confounding factors on the effect.

## 7 CONCLUSION

Although the self‐management interventions we include in the literature are different, the main symptoms are around management, behaviour management, drug management and psychological support. Four aspects for patients provide comprehensive, standardized or individualized instruction, with regular telephone follow ‐ up supervision and at the same time, improved the prognosis of patients with chronic heart failure. By evaluating and helping patients develop personalized self‐management interventions, medical staff can teach patients to recognize the symptoms and changes of the disease, guide patients to adopt correct behaviour and lifestyle management, and provide them with support and psychological counselling. In conclusion, self‐management intervention reduces the readmission rate of patients with chronic heart failure, improves their self‐management ability and quality of life, but the impact on mortality needs further research and exploration.

## AUTHOR CONTRIBUTIONS

QZ and SQZ conceived the idea and wrote the manuscript with input from CQF, SL prepared the figures and YMW prepared the tables. All the authors read and approved the final manuscript to be published.

## FUNDING INFORMATION

This work was supported by Henan Province's key R&D and promotion projects (scientific and technological research) projects (No. 222102310587), Key Scientific Research Project Plan of Henan Province (No. 22A310011) and Yellow River Scholar Foundation of Henan University for Shanqing Zheng.

## CONFLICT OF INTEREST

The authors declare that the research was conducted in the absence of any commercial or financial relationships that could be construed as a potential conflict of interest.

## ETHICAL APPROVAL

This article does not contain any studies with human participants or animals performed by any of the authors.
